# Peritransplant Varicosis After Simultaneous Pancreas and Kidney Transplantation Is an Uncommon Cause of Late-Onset and Recurrent Gastrointestinal Bleeding

**DOI:** 10.7759/cureus.40522

**Published:** 2023-06-16

**Authors:** Matthias Pfister, Adrian Kobe, Thomas Pfammatter, Marco Bonani, Fabian Rössler

**Affiliations:** 1 Surgery and Transplantation, Universitatsspital Zürich, Zürich, CHE; 2 Diagnostic and Interventional Radiology, Universitätsspital Zürich, Zürich, CHE; 3 Nephrology, Universitätsspital Zürich, Zürich, CHE; 4 Surgery and Transplantation, Universitätsspital Zürich, Zürich, CHE

**Keywords:** gastrointestinal bleeding, peritransplant varicosis, simultaneous pancreas and kidney transplantation, pancreas transplantation, • kidney transplantation

## Abstract

Ectopic peritransplant varicosis represents an uncommon cause of late-onset gastrointestinal (GI) bleeding after simultaneous pancreas and kidney transplantation (SPK). We report on a 53-year-old female patient who suffered from recurrent upper GI bleeding seven years after SPK with persistent graft function. Upper endoscopy revealed perianastomotic angiodysplasias, treated by clipping and Argon-Plasma-Coagulation. Repeated endoscopy showed no signs of anastomotic ulcer. With persistent symptoms, computed tomography and angiography revealed extensive ectopic varicosis around the pancreas and duodenal graft. With no signs of portal hypertension, pancreas graft venous outflow impairment or arterio-venous fistula, the origin of variceal formation remained unknown. The extended finding did not allow for endovascular treatment by embolization. Surgery with extensive variceal ligation led to persistent cessation of hemorrhage and maintained stable graft function. In patients with unclear recurrent upper GI bleeding after SPK, one should consider ectopic peritransplant varicosis as an exceptional bleeding cause. If endoscopic treatments fail, angiography should be performed to rule out unusual causes of vascular complications. In case of extensive peritransplant varicosis, surgery may remain the only successful therapy, whenever possible including graft preservation in well-functioning grafts.

## Introduction

Simultaneous pancreas and kidney transplantation (SPK) remains the benchmark treatment in selected patients with type-I diabetes mellitus (DM I) and chronic kidney disease (CKD), with excellent long-term patient and graft survival and high rates of insulin independence [1,2]. SPK is a technically challenging procedure complicated by the recipients' high vascular risk profile associated with poorly controlled DM I over many years. Vascular complications, together with graft pancreatitis and duodenal leakage, remain the most common causes of morbidity. However, graft thrombosis represents the main reason for non-immunologic graft failure after SPK [3].

In contrast, gastrointestinal bleeding (GI) after SPK is less frequent. Late onset of hemorrhage is mostly related to anastomotic ulcers of the graft duodeno-enterostomy, or severe vascular malformations like arterio-enteric fistula or pseudoaneurysms [4,5]. An even rarer cause of intestinal ulcers may be a gastrointestinal cytomegalovirus infection, which can particularly occur in immunosuppressed patients [6]. However, ectopic peritransplant varices as a source of late GI bleeding are overall rare and challenging to diagnose and treat. Few reports exist on variceal bleeding in patients after SPK, and mostly in patients with portal hypertension. No matter the cause of bleeding, management remains challenging for technical reasons. Endoscopy may be helpful, although the major limitation of a duodeno-jejunostomy is the limited access for standard endoscopy to visualize the anastomosis, and push enteroscopy is needed. Endovascular interventions might help in the treatment of arterio-venous fistulas [7,8] or pseudo-aneurysms [9], but to date no successful case has been reported for hemorrhage due to peripancreatic varices after SPK. However, surgery with graft pancreatectomy remains the ultima ratio in patients with nonfunctioning grafts, or in extreme cases with massive shocking bleeding. Nevertheless, graft preservation should remain a priority in patients with a well-functioning graft.

Here we report on a rare case of late recurrent GI bleeding after SPK due to prominent ectopic peritransplant varices, and without the presence of portal hypertension or venous outflow obstruction. We highlight the diagnostic and therapeutic challenges associated with bleeding complications after SPK and present successful graft-preserving surgical management of ectopic peritransplant variceal bleeding eight years after transplantation.

## Case presentation

Our patient, a 53-year-old woman, underwent SPK in 2014. Intra- and postoperative course was uneventful, with immediate and persistent graft function. Previous history was diabetes mellitus type 1, diagnosed at the age of 27, with unstable glycemia and repeat hypoglycemic episodes, as well as severe diabetic nephropathy present at the time of diagnosis. At the time of transplantation serum creatinine was 493 µmol/l, equal to a glomerular filtration rate (GFR) of 9 ml/min according to the Chronic Kidney Disease Epidemiology Collaboration (CKD-EPI) equation [10]. Both grafts were from a 20-year-old male donor after brain death (DBD) with traumatic brain injury. Pancreas was procured en bloc with duodenum and spleen, and transported by static cold storage. On the back table, a donor iliac Y-graft, connecting the superior mesenteric and splenic artery of the pancreas, was prepared. After the midline incision, at first, the pancreas graft was implanted. Venous drainage was systemic, via graft portal vein to the inferior vena cava. Arterial anastomosis was via Y-graft to the right common iliac artery. Exocrine drainage was enteric via graft duodeno-jejunostomy. The kidney was grafted to the left external iliac vessels. The total duration of surgery was 420 minutes, with a cold-ischemia time of 448 minutes for the pancreas and 608 minutes for the kidney. Intraoperative and short-term postoperative course was uneventful. Graft function was immediate and excellent, with persistent insulin freedom since transplantation and HbA1c levels below 5.5%.

Seven years after SPK, the patient developed GI bleeding for the first time. By that time, the patient was on Rivaroxaban (Xarelto®) for hereditary thrombophilia with deep vein thrombosis. Externally performed gastroscopy showed no pathology, however the graft duodeno-jejunostomy was not accessible for examination. Colonoscopy revealed possible signs of previous diverticular bleeding, and capsule endoscopy was inconclusive due to fecal contamination and delayed passage due to diabetic enteropathy. After the initial bleeding stopped and anticoagulation was discontinued, GI bleeding recurred with the persistent need of 1-2 erythrocyte (EC) transfusions every month. Coincidentally, kidney function and general health status slowly deteriorated over time. Recurrent erythropoietin and iron infusions were administered for severe anemia. After one year, a second capsule endoscopy revealed angiodysplasias at the site of graft duodeno-jejunostomy. Thereon push enteroscopy with Argon-Plasma-Coagulation (APC) was performed. Two days after the intervention, the patient suffered from hemorrhagic shock and emergency push enteroscopy revealed severe iatrogenic anastomotic bleeding, which was successfully treated with endoscopic clips and APC.

**Figure 1:**
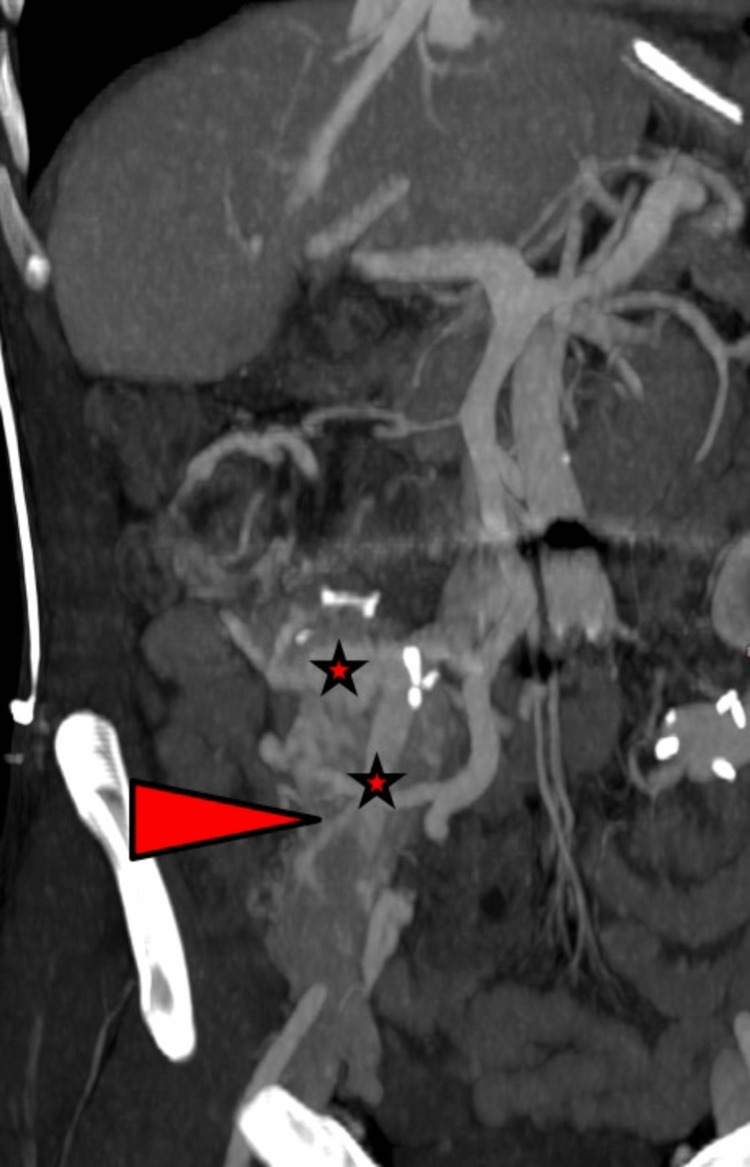
Computed tomography shows extended varicose veins around the pancreas and duodenal graft Arrow-head: pancreatic graft Asterisks: peritransplant varices

Two months later, during holidays abroad, the patient was admitted to the local emergency department for severe hematemesis with hemorrhagic shock and need for mass EC transfusion. Emergency upper and lower endoscopy were without result, but no push enteroscopy was available there. After repatriation to Switzerland, computed tomography (CT) showed no signs of recurrent bleeding, however, several pathologically enlarged vein branches around the pancreas and duodenal graft were seen (Figure 1).

**Figure 2:**
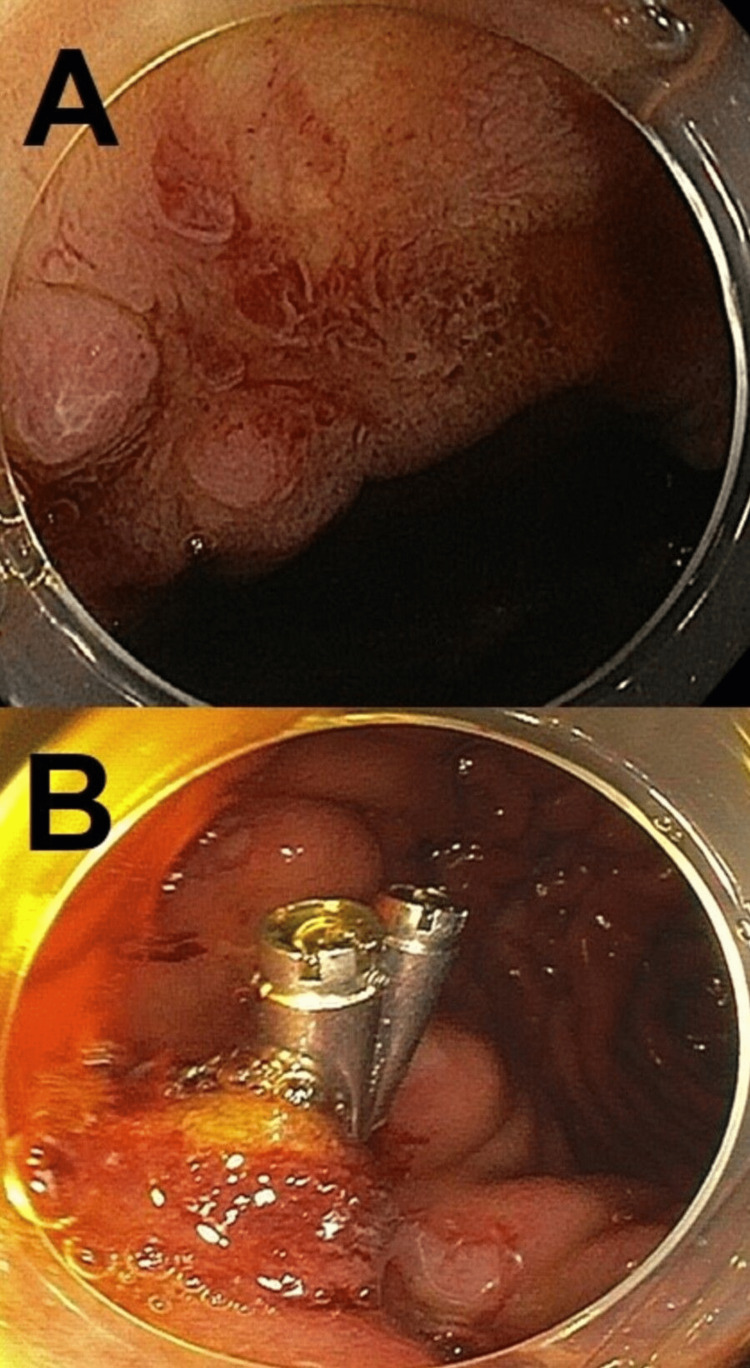
Push enteroscopy showed several areas of neovascularization (A), treated by Argon-Plasma-Coagulation and clipping (B).

There was no sign of anastomotic portal vein stenosis or thrombosis. Push enteroscopy revealed recurrent neovascularizations at the site of duodeno-jejunostomy, which were treated by clip application and adrenalin injections (Figure 2).

**Figure 3:**
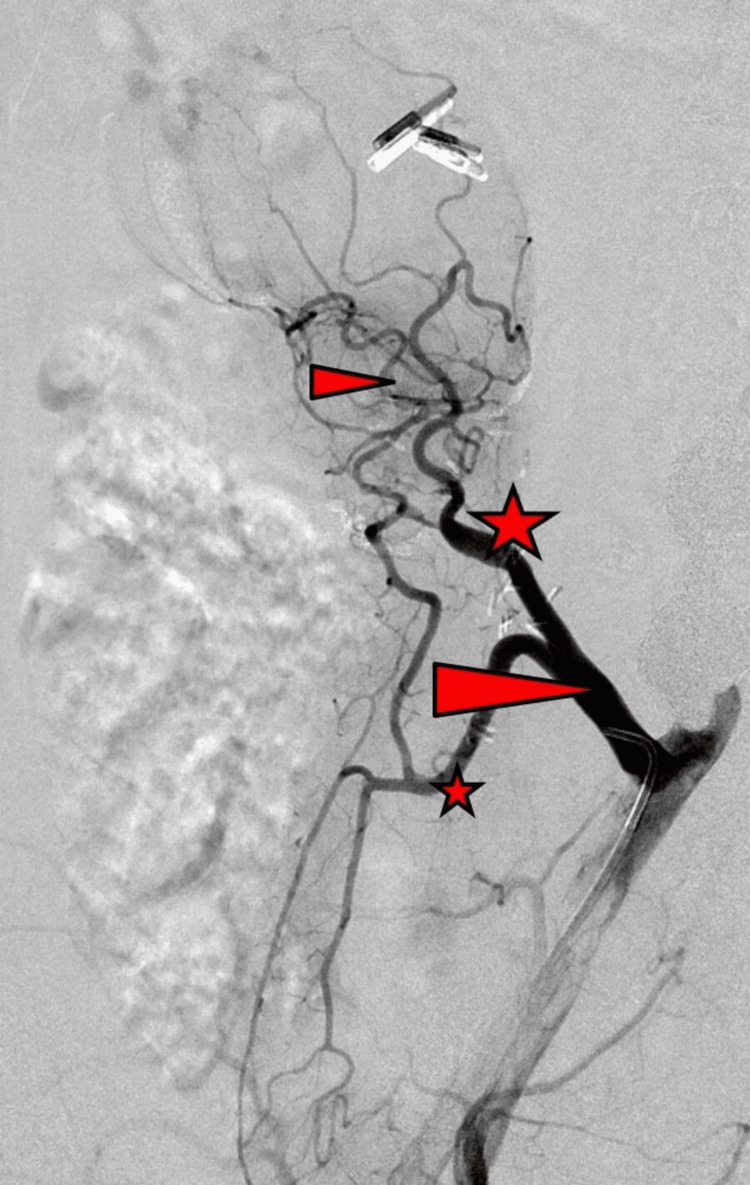
Arterial angiogram shows a regular arterial finding, without signs of arterio-venous fistula or pseudoaneurysm and a normal Y-patch. Large arrow-head: arterial Y-Graft anastomosed to the right common iliac artery Small arrow-head: arterial arcade around the graft pancreatic head and duodenum Large asterisks: graft superior mesenteric artery Small asterisks: graft splenic artery

There was no sign of graft rejection, with persistent insulin freedom and without elevation of serum amylase levels. That's why we decided against graft biopsy due to the severe risk of bleeding. Decision was made to undergo graft angiography to reveal the cause of the extended variceal formation. Arterial angiography was without pathological finding, excluding an arterio-venous fistula or pseudoaneurysm (Figure 3).

**Figure 4:**
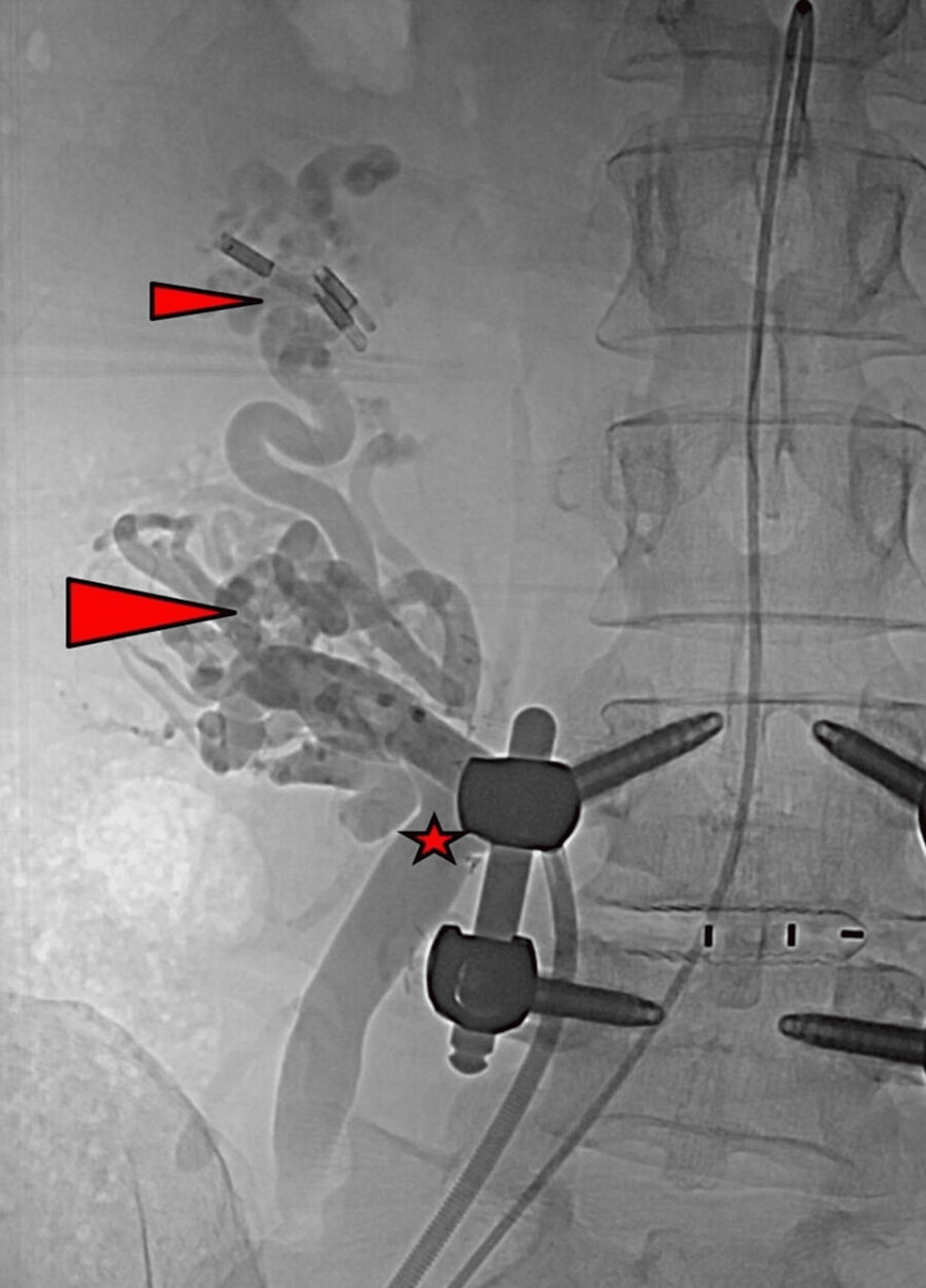
Venous angiogram shows peritransplant variceal and tortuous veins with endoscopic clip material. Large arrow-head: varicose veins around the graft pancreatic head Small arrow-head: varicose veins around the graft duodenum with endoscopic clips Asterisks: graft venous outflow via graft portal vein to the distal inferior vena cava

Venous angiography revealed numerous veins, also variceal and tortuous veins, around the pancreatic head and duodenal graft draining into the portal vein (Figure 4).

One varix was identified to even drain via mesentery to recipients' portal vein. Graft venous outflow was not impaired, with rapid venous drainage via graft portal vein into the inferior vena cava. Due to these extensive findings of peritransplant varicosis we refrained endovascular embolization due to the little expected chance of success. In light of persistent venous thrombosis in the left external iliac vein with the risk of kidney graft involvement, therapeutic anticoagulation with Phenprocoumon (Marcoumar®) was maintained.

**Figure 5:**
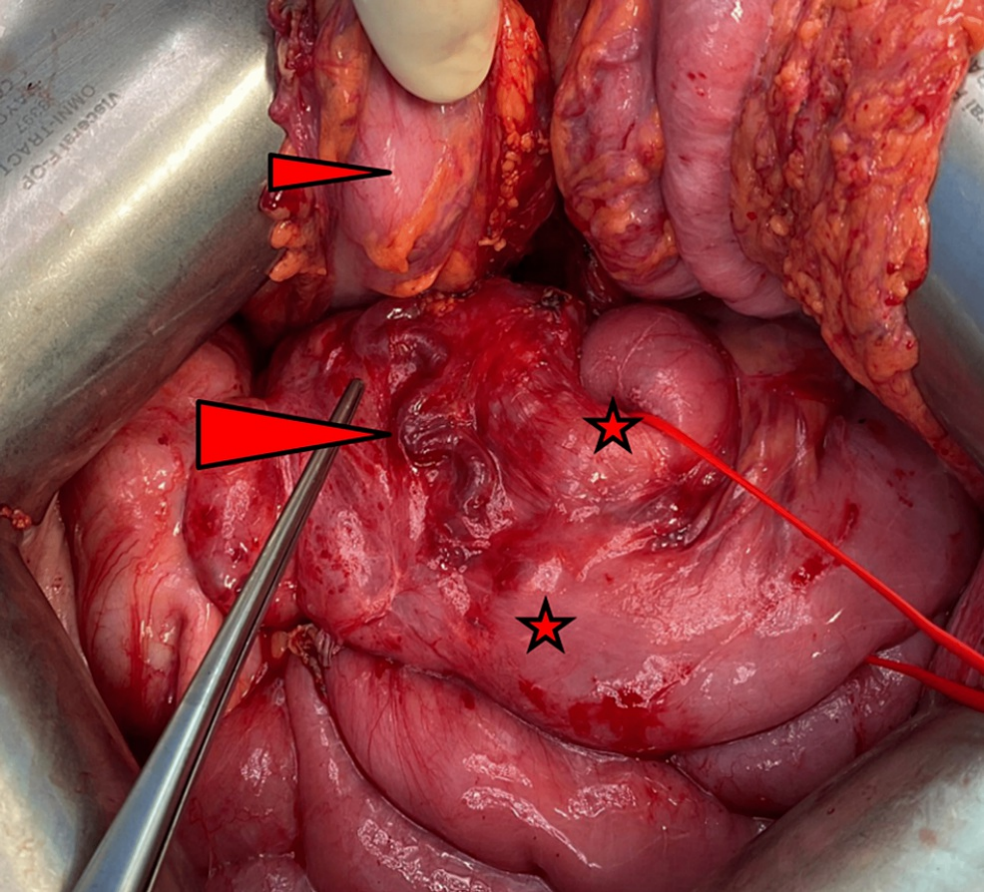
Intraoperative image of peritransplant varicosis Large arrow-head: large varicose vein on the graft duodenum and the anastomosed proximal jejunum Small arrow-head: large bowel Asterisks: proximal jejunum, anastomosed to the graft duodenum

With ongoing episodes of GI bleeding, unsuccessful endoscopic treatment and little chance for success of endovascular treatment, interdisciplinary panel discussion opted for surgical exploration. 8.5 years after SPK, we performed median laparotomy. Around the pancreatic head and the duodenal graft, numerous varicose veins were found (Figure 5).

Pancreatic and duodenal grafts were otherwise normal and the site of enteric anastomosis was without abnormalities. Numerous varicose veins were ligated. Intra- and postoperative course was uneventful and there was no sign of graft venous congestion after these ligations. Since surgery, the patient had complete resolution of symptoms and no more episodes of gastrointestinal bleeding occurred within eight months of follow-up. Graft function was not compromised by surgery as insulin freedom was maintained, and renal function remained stable.

## Discussion

This report describes a case of extensive peritransplant varicosis several years after SPK, causing recurrent severe upper GI hemorrhage, in a patient without portal hypertension, venous outflow obstruction or arterio-venous fistula. After failed endoscopic treatments and without the possibility for endovascular therapy, surgery with variceal ligation was finally successful, with sustained cessation of hemorrhage and persistent pancreas graft function. To the best of our knowledge, this is the first case of graft-preserving surgery for recurrent varicose bleeding after SPK. This case highlights the challenges in the management of vascular complications after SPK and illustrates the importance of an interdisciplinary team approach.

Late GI bleedings after SPK are overall uncommon and may occur even several years after transplant. These are usually either caused by severe vascular malformations such as arterio-enteric fistulas and pseudoaneurysms, or graft rejection or infection [4,5]. With the growing use of enteric versus bladder drainage in SPK within the last decades, the number of GI bleedings increased [11]. As most enteric drainages are via graft duodeno-jejunostomy, these anastomoses are difficult to assess by standard endoscopy and require push enteroscopy. This, in turn, bears the increased risk of ischemic anastomotic ulcer formation and possible consecutive duodenum graft perforation associated especially with recurrent endoscopic clipping and APC applications. An uncommon cause of GI bleeding after pancreas transplantation is arterio-enteric fistula [7,12]. This complication is often a sequence of enteric leakage and often graft pancreatectomy remains the only treatment option [12].

Variceal bleeding as a cause of recurrent GI bleeding after SPK is overall rare and to date, only few cases have been reported. Importantly, most reported cases occurred in patients with either portal hypertension [13] or with impaired venous outflow, like graft portal vein stenosis [14,15]. Only one report exists on a patient with peritransplant varicosis without signs of portal hypertension [14]. However, in this case, GI bleeding occurred earlier, already one year after transplantation, and the patient had previously suffered from a peritransplant abscess treated by percutaneous drainage [14].

However, in our case, the cause of peritransplant varicose vein formation remains unclear. Although portal venous pressure was not measured, there were no indirect signs of liver disease or portal venous hypertension in this patient. Graft portal vein stenosis was excluded by angiography. However, this does not exclude possible damage or compromise of smaller veins within the graft, which is impossible to detect. Other possible triggers of neovascularization, like peritransplant infection, graft pancreatitis, severe rejection, or infectious causes such as cytomegalovirus, were not present in our patient. Formation of varicosis subsequent to extensive endoscopic clipping and APC application is rather unlikely due to the short time interval between endoscopy and CT. However, only one CT scan was performed right before venous angiogram, so there is no possibility for comparison of the variceal formation and size over time. Most likely, variceal formation was triggered by surgical preparation compromising small peripheral venous outflow. Such formation of jejunal varicosis had been previously described in patients after hepatopancreatobiliary surgery [16,17], and was treated successfully by endovascular embolization and transhepatic portal vein stenting [16]. An additional influence may have been the effect of long-standing diabetes on neoangiogenesis, as DM I has been associated with excessive or defective new vessel formation [18].

Overall, treatment of variceal formation after SPK is challenging and to date, no recommendations or guidelines exist. Endovascular therapy plays a pivotal role in the treatment of vascular complications after SPK, above all arterio-venous fistula [7,8], graft thrombosis [19,20] or pseudoaneurysms [9]. However, to date, data on endovascular embolization of peritransplant varicosis is scarce and mostly relies on individual reports [13,14]. Fontana et al. reported on a successful percutaneous embolization of a mesenteric varix in a patient after SPK and liver transplantation [13]. This patient suffered from graft cirrhosis with portal hypertension, which led to the development of a mesenteric-caval shunt through the duodenum of the pancreas graft [13]. Rostambeigi et al. [14] reported complete variceal obliteration after embolization with ethylene alcohol (Onyx®). However, this did not prevent from further GI bleedings and consecutive salvage surgery was necessary.

In our case, after interdisciplinary discussion, we opted against endovascular variceal embolization. Because of the extent of the finding, with varices both around the graft duodenum and the adjacent jejunum, not only a transpancreatic but also a transhepatic endovascular procedure would have been required for embolization. The significantly increased risk of intestinal congestion or ischemia associated with such an extensive procedure, with only little chance for success in light of numerous and large varices, was ultimately considered unbearable. Importantly, in our patient, angiography could exclude other vascular complications such as arterio-enteric fistula or pseudoaneurysm as causes of bleeding. In our opinion, angiography is of utmost importance in patients with hemorrhage that cannot be treated endoscopically to rule out such complex vascular complications after SPK.

In exceptional cases with severe hemorrhage, graft pancreatectomy represents the only therapeutic option [14,15]. Most catastrophic vascular complications, such as arterio-venous fistulas, occur in nonfunctioning grafts [8]. The decision to remove a nonfunctional graft is less problematic. However, to date, no report exists on graft-preserving surgery for recurrent varicose bleeding. In our case, the graft function was excellent. Hence, our main goals were first to prevent future bleedings and second to preserve pancreas graft function. Given the possible surgical morbidity and the potential need to explant a functioning pancreas allograft due to intraoperative complications, the decision to undergo surgery was taken by an interdisciplinary team, involving surgeons, interventional radiologists and gastroenterologists. Reoperation of patients after SPK can be challenging and extensive variceal ligation might even lead to venous graft congestion and impaired function. In our case, surgery was uneventful and successful in regard to bleeding prevention and sustained graft function.

## Conclusions

In conclusion, this report describes a rare case of peritransplant variceal bleeding after SPK in a non-cirrhotic patient with preserved graft function in the absence of impaired graft venous outflow, or other complex vascular complications. After failed treatment attempts including recurrent advanced endoscopies and with endovascular therapy considered unfeasible, surgery with variceal ligation was successful. One should consider newly formed ectopic peritransplant varices as a rare cause of late GI bleeding after SPK. Patients with pancreas transplants, where GI bleeding cannot be localized or treated by endoscopy, should undergo angiography to exclude this uncommon etiology.
